# Levels of L-malate and other low molecular weight metabolites in spores of *Bacillus* species and *Clostridium difficile*

**DOI:** 10.1371/journal.pone.0182656

**Published:** 2017-08-29

**Authors:** George Korza, Stephen Abini-Agbomson, Barbara Setlow, Aimee Shen, Peter Setlow

**Affiliations:** 1 Department of Molecular Biology and Biophysics, UConn Health, Farmington, CT, United States of America; 2 Department of Molecular Biology and Microbiology, Tufts University School of Medicine, Boston, MA, United States of America; University of Groningen, NETHERLANDS

## Abstract

Dormant spores of *Bacillus* species lack ATP and NADH and contain notable levels of only a few other common low mol wt energy reserves, including 3-phosphoglyceric acid (3PGA), and glutamic acid. Recently, *Bacillus subtilis* spores were reported to contain ~ 30 μmol of L-malate/g dry wt, which also could serve as an energy reserve. In present work, L-malate levels were determined in the core of dormant spores of *B*. *subtilis*, *Bacillus cereus*, *Bacillus megaterium* and *Clostridium difficile*, using both an enzymatic assay and ^13^C-NMR on extracts prepared by several different methods. These assays found that levels of L-malate in *B*. *cereus* and *B*. *megaterium* spores were ≤ 0.5 μmol/g dry wt, and ≤ 1 μmol/g dry wt in *B*. *subtilis* spores, and levels of L-lactate and pyruvate in *B*. *megaterium* and *B*. *subtilis* spores were < 0.5 μmol/g dry wt. Levels of L-malate in *C*. *difficile* spores were ≤ 1 μmol/g dry wt, while levels of 3PGA were ~ 7 μmol/g; the latter value was determined by ^31^P-NMR, and is in between the 3PGA levels in *B*. *megaterium* and *B*. *subtilis* spores determined previously. ^13^C-NMR analysis of spore extracts further showed that *B*. *megaterium*, *B*. *subtilis* and *C*. *difficile* contained significant levels of carbonate/bicarbonate in the spore core. Low mol wt carbon-containing small molecules present at > 3 μmol/g dry spores are: i) dipicolinic acid, carbonate/bicarbonate and 3PGA in *B*. *megaterium*, *B*. *subtilis* and *C*. *difficile*; ii) glutamate in *B*. *megaterium* and *B*. *subtilis*; iii) arginine in *B*. *subtilis*; and iv) at least one unidentified compound in all three species.

## Introduction

Spores of *Bacillus* species normally have minimal if any metabolic activity and are extremely resistant to a wide variety of harsh treatments [1]. As a consequence, spores can survive for years in the absence of nutrients. However, given the proper stimulus, generally the presence of appropriate nutrients, spores can rapidly return to life in the processes of germination and outgrowth, and then resume vegetative growth [2,3]. Reflective of their metabolic dormancy, these spores have minimal if any levels of common intracellular low mol wt high energy compounds in their central core, including ATP and other nucleoside triphosphates, reduced pyridine nucleotides and acyl-CoA derivatives [1,4]. However, AMP and other ribonucleoside monophosphates, oxidized pyridine nucleotides and CoA are present in spores at levels similar to those in growing cells. In addition, spores contain several endogenous low mol wt energy reserves, which could be used to generate ATP, NADH, NADPH and acyl-CoAs soon after spore germination is initiated [2,4,5]. These potential energy reserves include: i) 3-phosphoglyceric acid (3PGA), which is rapidly catabolized to acetate following initiation of spore germination (although acetate is normally not catabolized further by outgrowing spores, which lack enzymes of the tricarboxylic acid cycle); and ii) significant levels of free amino acids most notably arginine and glutamate and at least some amino acid catabolic enzymes [5–8]. Another, and more significant energy reserve in dormant spores is the large amount of small, acid-soluble spore proteins in the spore core (> 5–10% of total spore protein) that are degraded to free amino acids early in spore outgrowth. Some of these amino acids are used for new protein synthesis in spore outgrowth, but much, along with spores’ large depot of free glutamate, are catabolized to generate energy or serve as precursors for other small molecules [9,10]. Overall, these endogenous spore core reserves of 3PGA and free and protein-bound amino acids are sufficient to support most ATP production and protein synthesis in the initial ~15 min following the initiation of spore germination, at least for *Bacillus megaterium* spores [5,9].

While spores’ endogenous reserves of amino acid and potential high energy compounds are significant, spores lack many other potential energy stores, in particular sugar-phosphates. L-lactate, pyruvate and mono-, oligo- or polysaccharides [4]. However, it was recently reported that dormant *B*. *subtilis* spores contain significant levels of L-malate, levels that were ~ 8 fold higher than those of 3PGA [11]. It was further suggested that this L-malate might be important in metabolism in the dormant spore to allow protein synthesis as one of the earliest steps in spore germination. Indeed, *Bacillus* spores are known to contain malate dehydrogenase that could oxidize L-malate to NADH plus oxaloacetate [7,12], although possible fates of oxaloacetate in dormant spores are not clear.

Although it is possible that spores could have large amounts of L-malate, ^13^C-NMR spectra of small molecules extracted from *B*. *subtilis* spores fail to reveal significant peaks that might be due to L-malate [13,14], although this was not noted in these studies. Enzymatic analysis of *B*. *megaterium* KM spore extracts for L-malate has also failed to detect significant L-malate levels [15]. Consequently, we have re-examined levels of malate in spores of three *Bacillus* species as well as *Clostridium difficile* with the intent of determining if this molecule does or does not play a significant role in metabolism in dormant or germinating spores. We have also determined the identity of almost all other major carbon-containing small molecules present at > 3 μmol/g dry wt in the core of *Bacillus megaterium* QM B1551, *Bacillus subtilis* and *C*. *difficile* spores.

## Materials and methods

### Spore-forming species used and spore preparation and purification

The spore forming species used in this work were *B*. *subtilis* PS533 [16], a prototrophic 168 laboratory strain, *B*. *megaterium* QM B1551 obtained from H.S. Levinson, *Bacillus cereus* T obtained from H.O. Halvorson, and *Clostridium difficile* CD630. Spores of these four species were prepared, purified and stored as previously described [17–21], and all spores used were free (> 98%) from growing or sporulating cells and germinated spores.

### Small molecule extraction from spores

Small molecules were extracted from dormant spores by several procedures. In Procedure 1 described a number of years ago [5], 1 ml of spores at an optical density at 600 nm (OD_600_) of ~ 80–200 was pipetted into 4 ml of boiling 1-propanol, the mix boiled for 5 min, cooled to ~ 23°C, flash evaporated, the dry residue extracted with several 2 ml aliquots of 4°C water followed by centrifugation in a microcentrifuge, and supernatant fluids were pooled and processed further prior to assays (see below). Previous work has shown that boiling 1-propanol treatment of growing cells or dormant spores gives excellent extraction and preserves even labile molecules such as ATP [5]. In some of these extractions various amounts of L-malate or other pure compounds (Sigma Chemical Company, St. Louis, MO) were added to spores just prior to mixing with boiling 1-propanol or just prior to NMR analyses to serve as internal standards, and to allow assessment of the recovery of L-malate and other compounds.

In Procedure 2, spores were extracted by mechanical disruption in liquid using a Mini-BeadBeater (Biospec Products, Bartlesville, OK) essentially as described previously [19]. In these extractions ~ 4–20 mg dry spores were shaken at room temperature with 0.75 g of 0.1 mm zirconium silica glass beads in 1 ml of 5 mM Tris-HCl buffer (pH 8.0) for 4 x 60 sec with cooling in between periods of shaking, followed by centrifugation in a microcentrifuge at top speed for 1 min, and the supernatant fluid was stored frozen. Spore breakage by this procedure was confirmed by microscopy as well as assays of dipicolinic acid (DPA) released from the spore core in the supernatant fluid, and this release was > 90%. In a few cases, the supernatant fluid was boiled for 20 min, centrifuged to remove coagulated protein, and the final supernatant fluid was stored frozen.

In one set of experiments, *Bacillus* spores were chemically decoated by incubation with sodium dodecylsulfate and dithiothreitol at alkaline pH for ~ 1 hr at 70°C (*B*. *subtilis*) or 60°C (*B*. *megaterium*), and washed extensively as described previously [22]. After buffer washes, decoated spores were washed once with 80% 1-propanol at room temperature for 2 min, and then washed several times with water. The decoated spores (50–60 mg dry wt) were then suspended in water, and extracted by Procedure 1 as described above. Finally, a mock extraction was run with 1 ml water and 4 ml boiling 1-propanol, and the dried material obtained after boiling was treated exactly as if it were a spore extract prepared in this manner.

### Assay of L-malate, L-lactate and pyruvate

L-Malate levels in spore extracts were determined in two ways. In one, spore extracts prepared by Procedure 1 were run through a Chelex column to remove Mn^2+^ ions that interfere with NMR analyses and then lyophilized, all as described previously [13,14,19]. The dry residue from lyophilization was dissolved in 700 μl D_2_O with 25 mM NaPO_4_ buffer (pH 7.4) for ^13^C-NMR, and 25 mM NaHepes buffer (pH 7.4) for ^31^P-NMR, and subjected to ^13^C-NMR or ^31^P-NMR analysis as described previously using 400 (^31^P) or 800 (^13^C) MHz instruments [13,14,19]. In some cases, small amounts of L-malate, L-lactate, pyruvate, acetate, formate, 3PGA, glutamic acid, arginine HCO_3_^-1^/CO_3_^-2^ or oxalic acid were added to NMR samples as internal standards to facilitate identification of various ^13^C-NMR peaks. Levels of small molecules were determined from intensities of NMR peaks of known amounts of pure compounds run in parallel with extracts as described previously [19]. ^13^C-NMR spectra of pure compounds run alone exhibited essentially identical peaks and peak heights seen with spore extracts to which these compounds were added. To validate assignment of various NMR peaks that were to be quantitated, known amounts of pure standards were added to spores at the beginning of extraction Procedure 1.

Lyophilized, Mn^2+^-free 1-propanol extracts prepared as described above were also assayed for L-malate enzymatically using malate dehydrogenase and monitoring NADH formation [11,23]. Extracts from spores prepared by mechanical disruption were also assayed enzymatically for L-malate. These enzymatic assays included multiple controls to ensure that the NADH that appeared to be derived from L-malate was indeed derived from this source, including assays: i) without added malate dehydrogenase; ii) using boiled extracts or directly from mechanically broken spores; and iii) with various amounts of pure L-malate added to serve as positive controls. L-Lactate and pyruvate in extracts from 1-propanol extracts of spores were also assayed enzymatically as described previously [6].

## Results

### Quantitation of L-malate and other organic acids in *Bacillus* spores by enzymatic analysis

Enzymatic analyses of spore extracts prepared by mechanical rupture in liquid were reported as showing that *B*. *subtilis* spores have very large amounts of L-malate, ~ 30 μmol/g dry spores [11]. There are, however, several concerns about this report as follows: i) published ^13^C-NMR spectra of extracts of *B*. *subtilis* spores fail to reveal significant peaks at the positions given by L-malate [13,14]; ii) previous work has not detected L-malate in spores of *B*. *megaterium* KM by enzymatic assays [2,15]; and iii) spore extracts prepared by mechanical rupture in liquid will have significant levels of many enzymes that are present in the spore core and are not inactivated during spore rupture, as well as many small and large molecules. In addition, the enzymatic assay for malate measures NADH production from L-malate catalyzed by malate dehydrogenase. Commercial malate dehydrogenase often has significant levels of lactate dehydrogenase, which could lead to erroneously high apparent levels of L-malate, which are in fact due to L-lactate, although L-lactate levels are reported to be extremely low in *B*. *megaterium* spores [6,15].

As a consequence of the concerns noted above, multiple controls are needed for unambiguous analysis of L-malate in crude extracts of bacterial cells or spores, especially if endogenous enzymes in extracts are not inactivated. Indeed, when such controls were done with such assays on extracts of *Bacillus* spores prepared by mechanical rupture in liquid (Procedure 2) (Table 1; and see Methods), no detectable L-malate was found in *B*. *cereus* or *B*. *megaterium* spores, consistent with previous results with *B*. *megaterium* spores [15]. However, a small amount of material reacting as L-malate (≤ 1–2 μmol/g dry spores) was detected by this assay in *B*. *subtilis* spores. Importantly, boiling of extracts prepared by Procedure 2 as soon as they were isolated, and then assaying for L-malate enzymatically gave the same results (Table 1). In addition to L-malate, spores of *B*. *megaterium* and *B*. *subtilis* lacked significant levels of L-lactate and pyruvate, as found previously for *B*. *megaterium* spores (Table 1) [6,15].

**Table 1:** Levels of organic acids in spores of various *Bacillus* species as determined by assays on spore extracts *.

Spores examined	L-Malate^2^	L-Lactate	Pyruvate
		μmol/g dry spores^1^	
*B*. *cereus*	< 1 (< 1)	nd^3^	nd^3^
*B*. *megaterium*	< 1 (< 1)	< 1^4^ (< 0.3)^5^	< 1^4^ (<0.05) ^5^
*B*. *subtilis*	≤ 1 (≤ 2)	< 1^4^ (< 0.5)^6^	< 1^4^ (< 0.5)^6^

*Spores of various species were extracted by Procedure 1 or 2 and small molecules were quantitated as described in Methods. For enzymatic assays of L-lactate and pyruvate in *B*. *subtilis* spores, all extracts were prepared by Procedure 1 but not passed through Chelex, but extracts were Chelex treated prior to ^13^C-NMR analyses.

^1^For comparative purposes, spores of *Bacillus* species have ~ 600 μmol of DPA/g dry spores [1].

^2^All values for L-malate were determined by enzymatic analyses. Values not in parentheses are from extracts made by Procedure 1, and values in parentheses are from extracts made by Procedure 2 and the supernatant fluid was boiled immediately after centrifugation as described in Methods.

^3^nd–not determined.

^4^Values determined by ^13^C-NMR.

^5^Data taken from reference 6 in which spores were extracted by a method similar to Procedure 1, and L-lactate and pyruvate were assayed enzymatically[15].

^6^Values in parentheses were determined by enzymatic assays in this work.

### Quantitation of L-malate in spores by ^13^C-NMR

**Fig 1:**
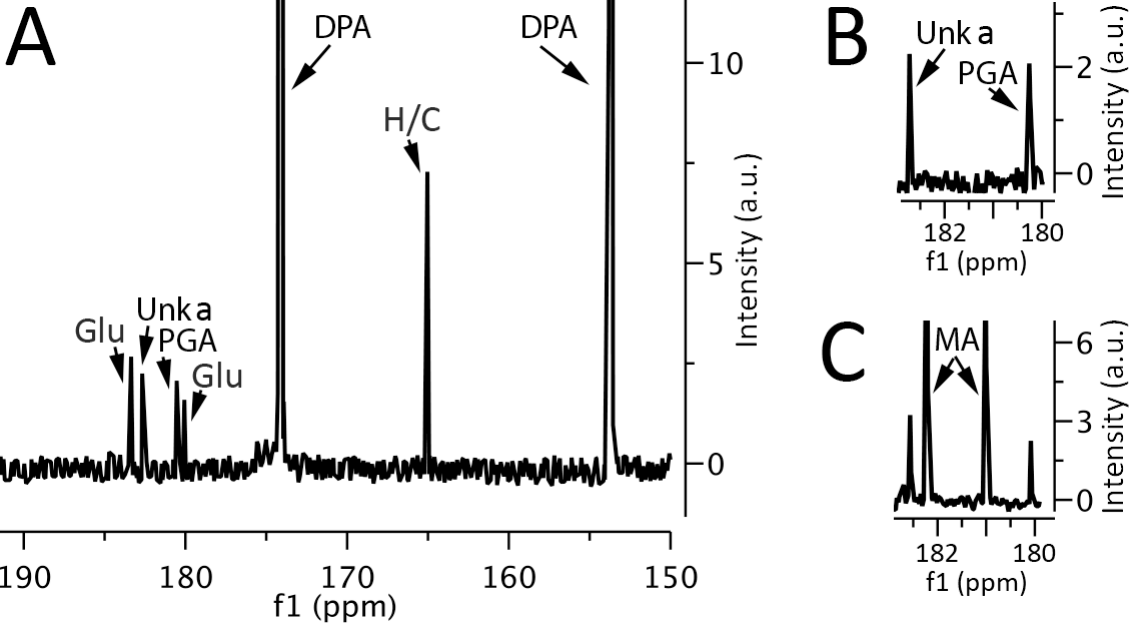
^13^C-NMR spectra of small molecules extracted from *B*. *megaterium* spores. Small molecules were extracted from ~ 65 mg dry wt of *B*. *megaterium* spores by Procedure 1 and extracts processed, ultimately dissolved in 700 μl D_2_O plus buffer and ^13^C-NMR spectra were collected as described in Methods. The various panels are the: A) ^13^C-NMR spectrum of the extract; B) expanded ^13^C-NMR spectrum of the extract in the region where peaks from L-malate would be expected; and C) expanded ^13^C-NMR spectrum of the extract as shown in B, but with 300 nmol L-malate added prior to extraction by Procedure 1. Peaks due to glutamate (Glu), 3PGA (PGA), HCO_3_^-1^/CO_3_^-2^ (H/C), malate (MA) and DPA are labeled, as is the peak given by an unknown compound (Unk a).

**Fig 2:**
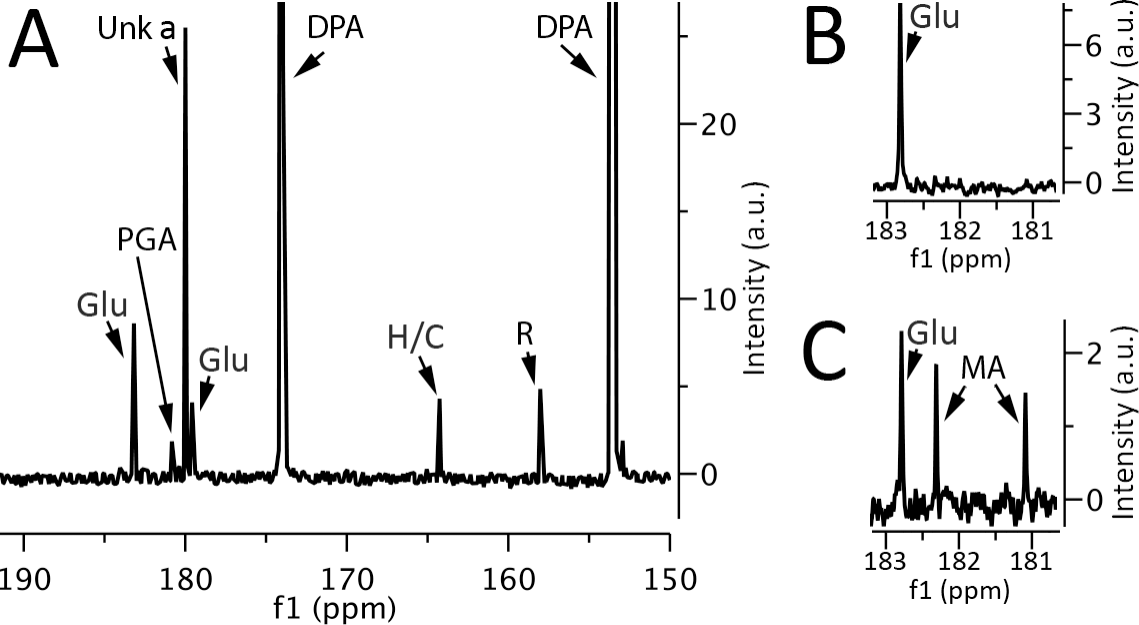
^13^C-NMR spectra of small molecules extracted from *B*. *subtilis* spores. Small molecules were extracted from ~ 130 mg dry spores of *B*. *subtilis* by Procedure 1 and extracts were processed, ultimately dissolved in 700 μl D_2_O plus buffer, and ^13^C-NMR spectra were collected as described in Methods. The various panels are the: A) ^13^C-NMR spectra of the extract; B) expanded ^13^C-NMR spectrum of the extract in the region where L-malate peaks would be expected; and C) expanded ^13^C-NMR spectrum of the extract shown in B but with 700 nmol of L-malate added prior to extraction by Procedure 1. Peaks due to glutamate (Glu), 3PGA (PGA), HCO_3_^-1^/CO_3_^-2^ (H/C), L-malate (MA), DPA and arginine (R) are labeled, as is the peak given by an unknown compound (Unk a).

While enzymatic assays indicated that a small amount of malate might be present, at least in *B*. *subtilis* spores, it was important to rigorously test this conclusion given the concerns about the enzymatic assay. Consequently, we turned to further ^13^C-NMR spectroscopy of concentrated spore extracts for both quantitation and identification of L-malate. These extracts were prepared by boiling spores with 1-propanol (Procedure 1), a procedure that rapidly inactivates enzymes in spores, and extracts high levels of labile small molecules such as ATP from growing bacteria or dormant or germinated spores [5]. Analysis of such *B*. *megaterium* spore extracts by ^13^C-NMR revealed no detectable peaks at the positions given by L-malate (Fig 1A–1C; Table 2) consistent with the minimal levels of L-malate detected by enzymatic assays of extracts made by mechanical rupture (Procedure 2) (Table 1). Indeed, enzymatic assays of Procedure 1 extracts from *B*. *megaterium* spores also gave no detectable L-malate, and enzymatic assays for L-malate in Procedure 1 extracts from *B*. *subtilis* spores gave less possible L-malate than determined by assays on extracts prepared by Procedure 2 (Tables 1 and 2). In addition, no peaks coincident with those given by L-malate were observed in ^13^C-NMR spectra of *B*. *subtilis* spore extracts prepared by Procedure 2 (Fig 2A and 2B). Notably, in control experiments in which known amounts of L-malate were mixed with spores just prior to extraction by Procedure 1, followed by processing of extracts and ^13^C-NMR, recoveries of added L-malate in *B*. *megaterium* and *B*. *subtilis* spore extracts were > 85% in two experiments, and the L-malate ^13^C-NMR peaks appeared at the expected positions with these doped samples (Figs 1C and 2C). Enzymatic assays of spiked extracts also yielded amounts of L-malate expected +/- 5%.

**Table 2:** Levels of L-malate, 3PGA and HCO3^-1^/CO3^-2^ in spores of various species as determined by ^13^C-NMR of 1-propanol extracts*.

Spores extracted	L-Malate	3PGA	HCO_3_^-1^/CO_3_^-2^
		μmol/g dry spores^1^	
*B*. *megaterium*	< 0.5 (< 0.5)^1^	27^2^	95
*B*. *subtilis*	< 1 (< 2)^1^	4^2^	55
*C*. *difficile*	< 1 (< 1)^1^	7	12

*Spores were extracted by Procedure 1, processed for NMR analyses and assayed for levels of L-malate and HCO_3_^-1^/CO_3_^-2^ by ^13^C-NMR and 3PGA by ^31^P-NMR. Values are all +/- ~ 20%.

^1^Values in parenthesis were determined by enzymatic assays of the sample analyzed by ^13^C-NMR.

^2^Values taken from reference 19, in which values were determined by ^31^P-NMR.

**Fig 3:**
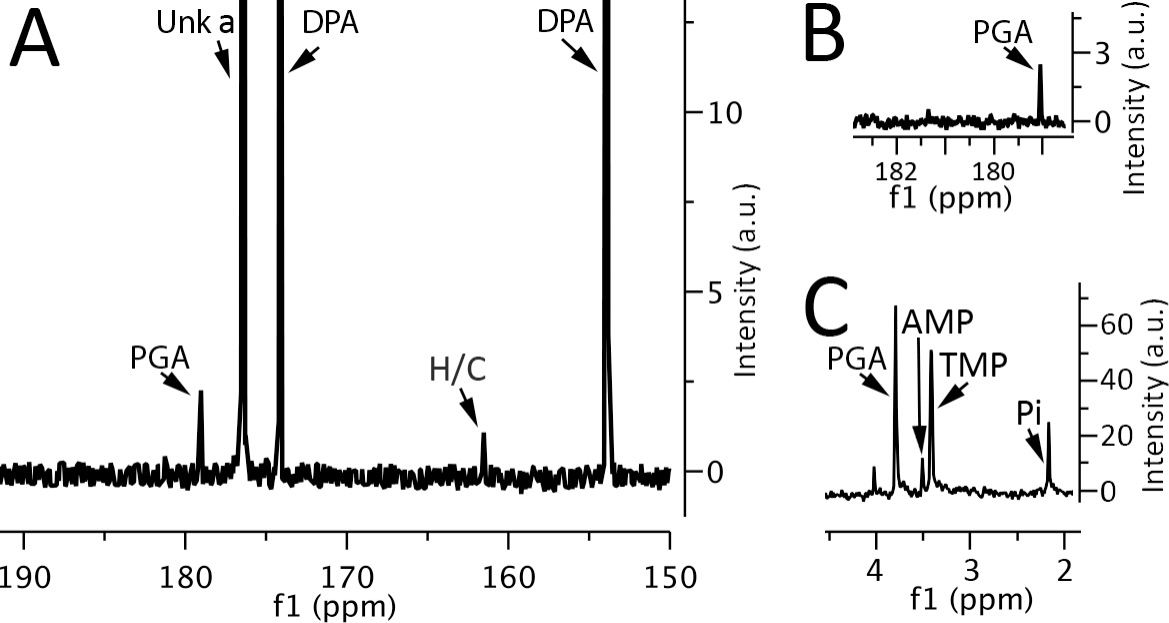
^13^C-NMR and ^31^P-NMR spectra of small molecules extracted from spores of *Clostridium difficile*. Spores of *C*. *difficile* strain CD630, ~ 35 mg dry wt, were extracted with Procedure 1, the extract processed, ultimately dissolved in 700 μl of D_2_O plus appropriate buffer, and NMR spectra were collected as described in Methods. The various panels are the: A) ^13^C-NMR spectrum of the extract; B) expanded ^13^C-NMR spectrum of the extract in the region where L-malate peaks would be expected; and C) ^31^P-NMR spectrum of the extract. In panels A,B) peaks due to 3PGA (PGA), HCO_3_^-1^/CO_3_^-2^ (H/C) and DPA are labeled, as is a peak given by an unknown compound (Unk a). In panel C) the identified peaks are PGA, AMP and inorganic phosphate (Pi). The TMP peak in panel C is from 175 nmol of thymidine-monophosphate added as a standard.

To further extend the analysis of L-malate to spores of *Clostridium* species, spores of *C*. *difficile* were extracted by Procedure 1 and analyzed by ^13^C-NMR. Again, no detectable peaks at the positions given by L-malate were found in these extracts (Fig 3A and 3B). In addition, enzymatic assay of the *C*. *difficile* extract revealed no detectable L-malate (Table 2).

### Levels of 3PGA in spores determined by ^31^P-NMR

While L-malate, L-lactate and pyruvate are absent from *B*. *megaterium* and *B*. *subtilis* spores and at least L-malate from *B*. *cereus* and *C*. *difficile* spores, spores of *Bacillus* species as well as at least *Clostridium bifermentans* and *Clostridium perfringens* contain significant levels of 3PGA (Table 2) [2,4,19,24–28]. An obvious question then is whether *C*. *difficile* spores also have 3PGA as an energy reserve for use during spore germination and outgrowth. ^31^P-NMR of *C*. *difficile* spore extracts indicated that these spores have only three major low mol wt phosphorylated molecules—inorganic phosphate, AMP and 3PGA (Fig 3C). The level of 3PGA in the *C*. *difficile* spores was also slightly higher than in *B*. *subtilis* spores, although lower than in *B*. *megaterium* spores (Table 2). *B*. *cereus* spores have also been reported to have 3PGA levels very similar to those of *B*. *megaterium* spores, as determined by analysis of small molecules in ^32^P-labeled spores [27,28].

### Identification of other small molecules in spores by ^13^C-NMR

While L-malate was not detected in ^13^C-NMR spectra of extracts from spores of *Bacillus* species, there were a number of other ^13^C-NMR peaks in these extracts (Figs 1 and 2). All these peaks are from molecules in the spore core since: i) ^13^C-NMR spectra of extracts made by Procedure 1 from chemically decoated spores gave approximately the same heights of the peaks in extracts from intact spores; and ii) the ^13^C-NMR spectrum of a mock spore extract made by Procedure 1 gave no peaks with > 2% of the intensities of peaks in spore extracts. Previous work has shown that in addition to 3PGA, spores of *Bacillus* species have very high levels of dipicolinic acid (DPA), and lower but significant levels of a few free amino acids, with glutamic acid by far the highest (28 and 70 μmol/g dry wt in *B*. *subtilis* and *B*. *megaterium* spores, respectively), much smaller amounts of arginine and even smaller amounts of lysine [8]. Analysis of the ^13^C-NMR spectra of Procedure 1 extracts from *B*. *megaterium* and *B*. *subtilis* spores with or without addition of known small molecules allowed identification of peaks from DPA, 3PGA, glutamic acid and arginine (Figs 1 and 2).

The ^13^C-NMR analyses described above left 2 significant peaks that were unassigned to known compounds in spores of *C*. *difficile* (small peak at ~163 ppm (H/C) and a very large peak at 177 ppm), *B*. *megaterium* (peaks at ~165 (H/C) and 184 ppm) and *B*. *subtilis* (peaks at 165 (H/C) and 180 ppm). One possibility was that one or both of these unidentified peaks in spore extracts might be due to some esterified derivatives of DPA that have been identified in low levels of spores of several species [29,30]. However, when spore extracts were adsorbed with sufficient activated charcoal to adsorb > 90% of their DPA, there was < 5% change in the intensity of the ^13^C-NMR peaks of unknown identity. Thus it seemed unlikely that the two unknown peaks are due to DPA derivatives.

The peak at ~ 165 ppm in ^13^C-NMR spectra of extracts from *B*. *megaterium*, *B*. *subtilis* and *C*. *difficile* spores is in the region of the single peak given by HCO_3_^-1^ and CO_3_^-2^, as the peaks from these two species coalesce into a single peak, whose precise location is determined by the pH of the sample. Indeed, addition of small amounts of Na_2_CO_3_ or NaHCO_3_ to *B*. *subtilis* spore extracts and subsequent ^13^C-NMR showed that the peak from the added HCO_3_^-1^/CO_3_^-2^ was superimposed on the peak at ~ 165 ppm. Thus this latter peak in spore extracts is most likely due to HCO_3_^-1^/CO_3_^-2^. This is one of the most the most abundant spore small carbon containing small molecules after DPA. However, the source of the likely HCO_3_^-1^/CO_3_^-2^ in the *Bacillus* spore core is not clear, and could even be derived from the mother cell compartment of the sporulating cell, which: i) generates much CO_2_ via action of the tricarboxylic acid cycle; and ii) has been suggested to supply mother cell molecules to the developing forespore by a “feeding tube” [31–33]. Much less putative HCO_3_^-1^/CO_3_^-2^ was present in the *C*. *difficile* spore core, consistent perhaps with this organism being an anaerobe, although this organism does have the capacity for at least amino acid decarboxylation. In contrast to the identification of the peak at ~ 165 ppm as most likely given by HCO_3_^-1^/CO_3_^-2^, the peak at ~ 180 ppm has not been identified, but our analyses suggest that it is not due to acetate, formate or oxalate, since the ^13^C-NMR peaks due to these latter compounds were not near 180 ppm in multiple experiments.

## Discussion

The results presented in this communication indicate that the major identified low mol wt metabolites in the central core of spores of *Bacillus* species as well as at least one *Clostridium* species are DPA, 3PGA, HCO_3_^-1^/CO_3_^-2^, and several free amino acids, with DPA levels much higher than all others put together. Significant levels of no other identified low mol wt compound containing carbon were detected in spore’s core, although spores of *B*. *megaterium*, *B*. *subtilis* and *C*. *difficile* did have quite significant levels of at least one unidentified carbon-containing compound. A number of low mol wt carbon-containing compounds, in particular 3PGA and glutamic acid, are catabolized soon after spore germination is initiated to provide energy for RNA synthesis and uptake of exogenous nutrients. Degradation of stored spore protein slightly after completion of germination will provide many more free amino acids that can be used for energy metabolism [5,6,9,10]. However, as far as is known, with germination in the absence of exogenous metabolites, dormant spores must rely only on stored 3PGA and free amino acids for generation of high energy compounds such as ATP and other ribonucleoside triphosphates immediately after germination is completed [5,6].

In this regard, in particular when the molecule L-malate is considered, the absence of significant malate in spores is consistent with previous work that did not detect this molecule in spores [2,12,15]. An obvious question is how did a previous study [11] find so much L-malate in spores? We do not know the answer to this question. However, possible answers are: 1) the malate detected was outside the spore core, although we did not detect L-malate in whole spores; or 2) overestimation of malate levels using an enzymatic assay for L-malate. A third possibility is that L-malate is actually generated enzymatically from a larger molecule in spore extracts, especially in extracts made in which no attempts were made to block enzyme activity during spore extraction or as soon as possible after extraction. One possible polymeric source of L-malate that could be envisaged is poly-L-malic acid, a polymer that has been found at significant levels in plasmodia of myxomycetes, along with enzymes for the synthesis and depolymerization of this polymer [34–37]. However, there are no reports of poly-L-malate in bacterial spores, nor have any genes for the synthesis and degradation of this polymer been identified in *Bacillus* species. In addition, no significant levels of L-malate were identified in spore extracts prepared by physical rupture of spores in liquid at pH 8. Significant L-malate levels were, however accumulated when *B*. *megaterium* spores were germinated with L-alanine, although the amounts were only ~ 1 μmol/g dry spores [15], 30-fold lower than reported recently in *B*. *subtilis* spores [11]. In addition, this latter L-malate accumulation was a bit slower than ATP accumulation in germination, and the accumulated L-malate could be generated by catabolism of amino acids generated by proteolysis of dormant spore protein. Overall, it appears most likely that dormant spores simply do not contain any large amount of L-malate, either free or polymerized, to serve as an energy source in dormant or germinating spores. However, dormant spores do have significant levels of other catabolites, in particular 3PGA, that can provide ATP and other high energy molecules soon after spore germination is initiated [5,6]. It is possible that this could also be the case for the major unidentified small molecule in spores, but like the likely HCO_3_^-1^/CO_3_^-2^, these molecules might also be simply sporulation “remnants”, although the HCO_3_^-1^/CO_3_^-2^, could play an important role in pH homeostasis in dormant and germinating spores. However, these possibilities are matters for future work.
